# PLAC8, a new marker for human interstitial extravillous trophoblast cells, promotes their invasion and migration

**DOI:** 10.1242/dev.148932

**Published:** 2018-01-15

**Authors:** Wen-Lin Chang, Ya-Wei Liu, Yan-Li Dang, Xiang-Xiang Jiang, Honglin Xu, Xing Huang, Yan-Ling Wang, Haibin Wang, Cheng Zhu, Li-Qun Xue, Hai-Yan Lin, Wenxiang Meng, Hongmei Wang

**Affiliations:** 1 State Key Laboratory of Stem Cell and Reproductive Biology, Institute of Zoology, Chinese Academy of Sciences, Beijing 100101, People's Republic of China; 2 Guangdong and Shenzhen Key Laboratory of Male Reproductive Medicine and Genetics, Institute of Urology, Peking University Shenzhen Hospital, Biomedical Research Institute, Shenzhen Peking University-The Hong Kong University of Science and Technology Medical Center, Shenzhen 518036, People's Republic of China; 3 College of Veterinary Medicine, Hunan Agricultural University, Changsha 410128, People's Republic of China; 4 University of Chinese Academy of Sciences, Beijing 100039, People's Republic of China; 5 Department of Obstetrics and Gynecology, the 306th Hospital of PLA, Beijing 100101, People's Republic of China; 6 State Key Laboratory of Molecular Developmental Biology, Institute of Genetics and Developmental Biology, Chinese Academy of Sciences, Beijing 100190, People's Republic of China

**Keywords:** PLAC8, Extravillous trophoblast cells, Marker, Invasion and migration, Placenta, Preeclampsia

## Abstract

Proper differentiation of trophoblast cells in the human placenta is a prerequisite for a successful pregnancy, and dysregulation of this process may lead to malignant pregnancy outcomes, such as preeclampsia. Finding specific markers for different types of trophoblast cells is essential for understanding trophoblast differentiation. Here, we report that placenta-specific protein 8 (PLAC8) is specifically expressed in the interstitial extravillous trophoblast cells (iEVTs) on the fetomaternal interface. Using model systems, including placental villi-decidua co-culture, iEVTs induction by using primary trophoblast cells or explants, etc., we found that PLAC8 promotes invasion and migration of iEVTs. Mechanistically, time-lapse imaging, GTPase activity assay, co-immunoprecipitation and RNA-seq studies show that PLAC8 increases the Cdc42 and Rac1 activities, and further induces the formation of filopodia at the leading edge of the migratory trophoblast cells. More interestingly, PLAC8 is significantly upregulated under hypoxia and expression of PLAC8 is higher in iEVTs from preeclamptic placentas when compared with those from the normal control placentas. Together, PLAC8 is a new marker for iEVTs and plays an important role in promoting trophoblast invasion and migration.

## Abstract (precis)

**Highlighted Article:** Oxygen tension-dependent expression of placenta-specific protein 8 positively regulates trophoblast invasion and migration partially through upregulating the activation of Rac1 and Cdc42.

## INTRODUCTION

The human placenta, a transient endocrine organ to connect the mother and the developing fetus for nutrient and gas exchange and immunological modulation, etc., is essential for embryonic development and the success of a healthy pregnancy (Murphy et al., 2006). The placental villus is the basic structural and functional unit of the placenta. Lining the placental villus is a layer of mononucleated cytotrophoblast cells (CTBs), which is overlaid by a continuous layer of multinucleated syncytiotrophoblast (STB) (Kaufmann et al., 1987). At the tip of the villus, CTBs proliferate and form a trophoblast cell column (TC) to break through the STB layer. At the distal region of the TC, trophoblast cells undergo an epithelial-to-mesenchymal transition (DaSilva-Arnold et al., 2015; Fu et al., 2010; Kokkinos et al., 2010; Vicovac and Aplin, 1996), thus gaining the motility characteristics typically needed to invade the maternal uterine wall (Kam et al., 1999). Trophoblast cells that invade the maternal decidua and anchor the placenta are called interstitial extravillous trophoblast cells (iEVTs), whereas the ones that remodel maternal spiral arteries to fulfill the increased maternal blood flow requirement of the placenta/fetus (Hunkapiller and Fisher, 2008; Pijnenborg et al., 2006) are termed endovascular EVTs (eEVTs) (Kam et al., 1999). eEVTs are further discriminated as intra-mural EVTs (imEVTs), which embed in the spiral artery wall or lining of the arterial lumen by replacing the arterial endothelial cells, and intra-artery EVTs (iaEVTs), which mainly plug the arterial lumen (Kaufmann and Castellucci, 1997). Abnormal EVT differentiation and subsequent shallow invasion into the decidua by iEVTs or insufficient remodeling of spiral arteries by eEVTs (called ‘utero-placental insufficiency’) are major contributors to the pathogenesis of preeclampsia (PE), a highly prevalent pregnancy-related complication characterized by new-onset hypertension and proteinuria after 20 weeks of gestation and causing maternal and perinatal morbidity and mortality (Zhou et al., 1993).

Finding proper markers to identify and separate different types of trophoblast cells is crucial for elucidating functions of trophoblasts and understanding the pathophysiology of pregnancy-related diseases (Harris et al., 2009; Kaufmann and Castellucci, 1997). Early anatomical studies of human placenta have identified a few trophoblast-specific markers or molecules (Chang et al., 2014; Kaufmann and Castellucci, 1997; Khong et al., 1986). Cytokeratin 7 (CK7) identifies all subtypes of human trophoblast cells at the fetomaternal interface (Kaufmann and Castellucci, 1997; Khong et al., 1986). Human leucocyte antigen G (HLA-G), the first EVT-specific molecular marker, is a non-classical major histocompatibility complex class I (MHC I) molecule that is expressed by EVTs so that they are able to evade attack by the maternal immune system (Ellis, 1990; Kovats et al., 1990). The integrin-switching and characteristic expression changes of immunoglobulin family cell-adhesion molecules (CAMs), i.e. selectins and cadherins, are very important for EVT differentiation (Harris et al., 2009). A unique polysialylated form of neural cell-adhesion molecule (NCAM) is expressed in the perivascular and plugged EVTs, but absent from villous trophoblasts and TCs (Burrows et al., 1994). Recently, leukocyte-associated immunoglobulin-like receptor 2 (LAIR2) was also revealed to be a possible EVT-specific molecule (Founds et al., 2010, 2013).

Placenta-specific protein 8 (PLAC8), a 12.5 kDa protein also known as ONZIN, is highly conserved from amphibians to humans. The homologous gene for mouse, *Plac8*, was first recognized as a placenta-specific transcribed gene (Galaviz-Hernandez et al., 2003). Subsequently, PLAC8 has been found to play important roles during cell apoptosis and proliferation as a c-Myc-repressed target (Mourtada-Maarabouni et al., 2013; Rogulski et al., 2005), and during the differentiation of acute myeloid leukemia cells (Wu et al., 2010). PLAC8 is also involved in tumorigenesis in pancreatic cancer (Kinsey et al., 2014) and in colon cancer (Li et al., 2014). *Plac8*^−/−^ mice exhibit deficiencies in innate immunity (Ledford et al., 2007), contact hypersensitivity (Ledford et al., 2012), and brown and white fat differentiation (Jimenez-Preitner et al., 2012, 2011); they also develop age-onset obesity (Blue et al., 2015; Sasaki et al., 2015). In the mouse placenta, *Plac8* mRNA is mainly localized in trophoblast giant cells at 6.5 and 8.5 dpc, and in spongiotrophoblast at 10.5 and 18.5 dpc, suggesting an important role for PLAC8 in placental development (Galaviz-Hernandez et al., 2003). In the human placenta, however, the function of PLAC8 remains elusive. In this study, we report that PLAC8 is a new marker for iEVTs and that oxygen tension-dependent expression of PLAC8 promotes invasion and migration of EVTs.

## RESULTS

### PLAC8 is exclusively expressed in the iEVTs of the human placenta

**Fig. 1.**
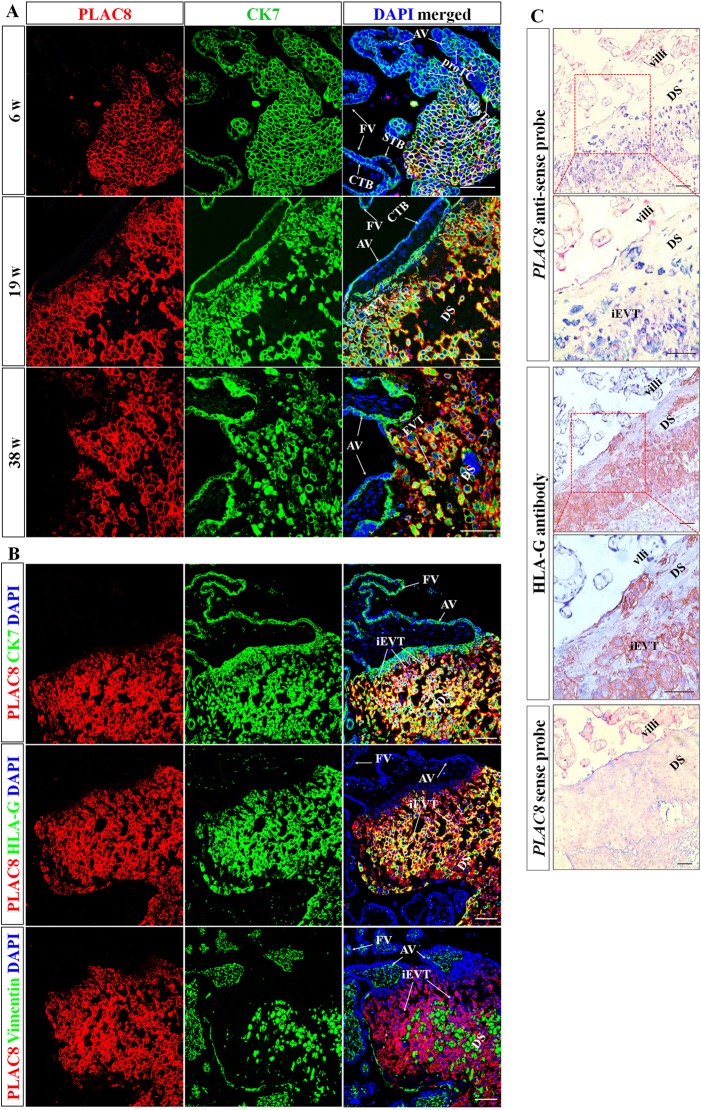
**PLAC8 is exclusively expressed in the human placental iEVTs.** (A) Immunofluorescent assessment of PLAC8 expression in sections of placental tissues from 6 weeks (first trimester, *n*=5), 19 weeks (second trimester, *n*=4) and 38 weeks (third trimester, *n*=3) of pregnancy using antibodies against CK7 and PLAC8. DAPI is used to stain the cell nuclei. AV, anchoring villi; FV, floating villi; proTC, proximal trophoblast cell column; disTC, distal trophoblast cell column; CTB, cytotrophoblast cells; STB, syncytiotrophoblast; DS, decidual side; iEVT, interstitial extravillous trophoblast cells. (B) Serial sections of placental tissues from second trimester placentas (*n*=4) were stained using the indicated antibodies. (C) *In situ* hybridization of the *PLAC8* mRNA on placental tissues from full-term gestation (*n*=3). An immunohistochemical assessment on a serial section was also performed using an anti-HLA-G antibody. Bottom panel, sense probe incubation on a serial section as a negative control of the *in situ* hybridization assay. Scale bars: 100 µm.

In an effort to elucidate whether placenta-specific protein 8 (PLAC8) plays a role in human placentation, we first sought to determine the expression pattern of PLAC8 in human placentas at different stages of pregnancy. Thus, we collected human placental villi at 6 weeks, 19 weeks and 38 weeks of pregnancy, representing placentas from the first, second and third trimesters, respectively, and performed immunofluorescent staining using antibodies against PLAC8 and cytokeratin 7 (CK7). As shown in Fig. 1A, PLAC8 was exclusively expressed in the trophoblast cell column (TC) in 6-week-old placental villi and an increased expression was detected from the proximal region of TC (proTC) to the distal region of TC (disTC). In 19-week-old and 38-week-old placental villi, specific PLAC8-positive staining was observed in the subpopulations of CK7-positive cells that were only assembled at the maternal side of the fetomaternal interface, which represent the interstitial extravillous trophoblast cells (iEVTs) that had invaded into the maternal decidua. However, no obvious PLAC8 immunostaining was detected in the villous cytotrophoblast cells (CTBs) or syncytiotrophoblasts (STBs) from all the three trimester placentas, which were also CK7-positive, implying that PLAC8 was highly expressed in only iEVTs in the human placentas.

To confirm this observation, we next performed immunofluorescent staining assays using antibodies against human leucocyte antigen-G (HLA-G), a specific molecular marker for extravillous trophoblast cells (EVTs). As shown in Fig. 1B, obvious PLAC8-positive staining was observed in the iEVTs that finely exhibited HLA-G-positive staining at the maternal side of the second trimester placental villi. Consistent data were obtained in the *in situ* hybridization assays (Fig. 1C), that the *PLAC8* mRNA was mainly localized in the iEVTs, as indicated by positive staining for the HLA-G antibody, whereas no specific positive signal was observed on the serial sections that were incubated with the *PLAC8* sense probe. As iEVTs undergo effective migration and invasion into the mother's uterus, we then used antibodies against vimentin to mark the uterine decidual cells. As shown in Fig. 1B, iEVTs that alternately localized in the crevices between vimentin-positive cells displayed strong PLAC8-staining signals, suggesting that PLAC8 expression is highly abundant in the iEVTs that have effectively invaded and migrated into the uterine wall and is absent in the maternal decidual cells.

To further test whether PLAC8 is definitely a unique marker for iEVTs in the whole placenta tissue, we obtained a broad view of PLAC8 expression pattern at the fetomaternal interface via a confocal tile scan picture consisting of 64 individual pictures that covered the whole 19 w placenta sections (0.5 cm×0.5 cm). As shown in Fig. S1, all the iEVTs displayed strong PLAC8 signals at the maternal side of the fetomaternal interface. Taken together, our data strongly suggest that PLAC8 was exclusively expressed in human placental iEVTs, but not other trophoblast subtypes, indicating that PLAC8 is a specific marker for iEVTs in the human placenta.

### PLAC8 is hardly detectable in the eEVTs

**Fig. 2.**
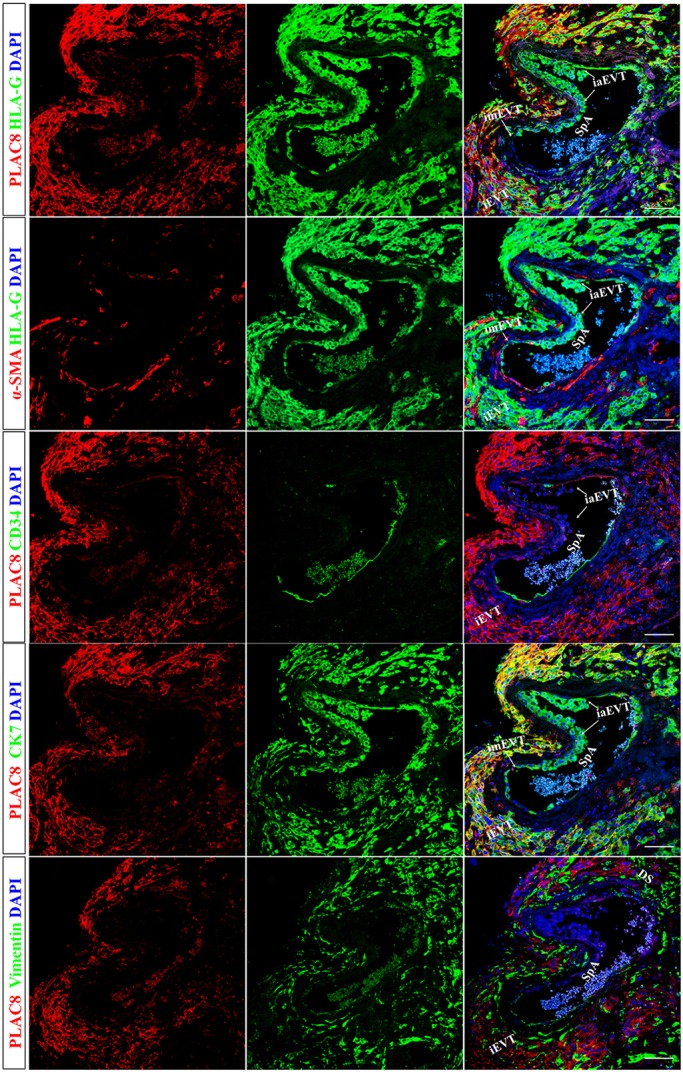
**PLAC8 is hardly detectable in the eEVTs.** Serial sections of a partially remodeled maternal spiral artery from the second trimester placental bed (*n*=4, a placenta of 19 weeks gestation age is shown here) were double-stained with indicated combinations of antibodies. iaEVTs, intra-artery EVTs; imEVTs, intra-mural endovascular EVTs; SpA, spiral artery. Scale bars: 100 µm.

Based upon the observations that PLAC8 was only abundant in iEVTs in placenta tissues, we then sought to determine the expression of PLAC8 in eEVTs, and again performed immunofluorescent staining assays. As shown in Fig. 2, serial paraffin sections of the fetomaternal interface with a partial remodeling spiral artery (see a schematic graph in Fig. S2) were double stained with antibodies against PLAC8 and HLA-G, HLA-G and α-SMA (the marker for smooth muscle cells), PLAC8 and CD34 (the marker for endothelium), PLAC8 and CK7, or PLAC8 and vimentin. We observed that the substantial loss of vascular smooth muscle cells and endothelium was compensated for by the *in situ* incorporation of EVTs, as illustrated by HLA-G and CK7 immunostaining (the first and fourth rows). The iaEVTs, which are positive for both HLA-G and CK7, can be seen in the lumen of the vessel, and some of these cells adhered to the vascular wall (the first, second and fourth rows). Of note, PLAC8 was barely detectable in the imEVTs and iaEVTs, which were strongly positive for HLA-G and CK7. However, in the iEVTs surrounding the spiral artery, strong PLAC8 signal was observed. These data strongly suggest that PLAC8 was highly concentrated only in iEVTs, but not in eEVTs.

### PLAC8 is induced during the differentiation of CTBs into iEVTs

**Fig. 3.**
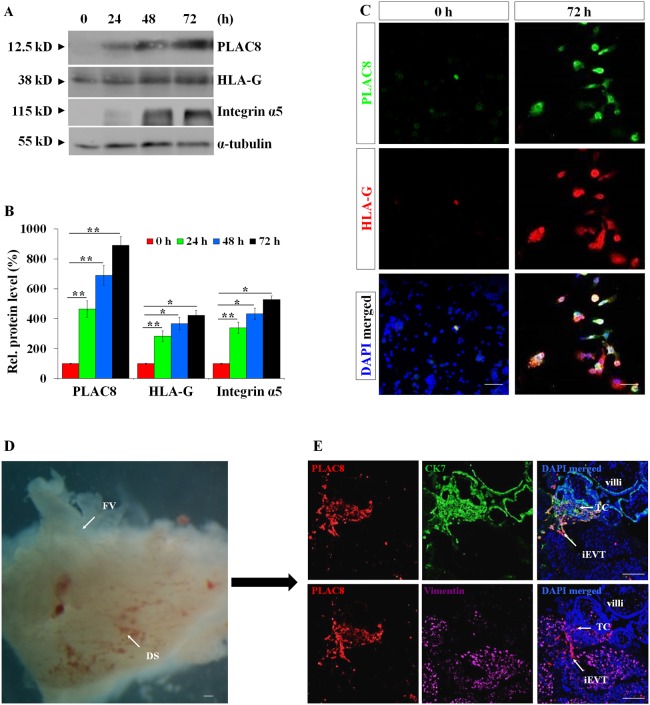
**PLAC8 expression is induced during the differentiation of CTBs into iEVTs.** (A) Successful iEVT differentiation from CTBs is indicated by the increased expression levels of the EVT markers HLA-G and integrin α5 by western blotting. α-Tubulin is a loading control. (B) Statistical analysis of the western blotting results representatively shown in A (*n*=5; *P<0.05, **P<0.01). (C) Immunofluorescent assessment of CTB cells (0 h and 72 h) using the indicated antibodies in the *ex vivo* EVT induction model. (D) Morphology of placental villi and decidua from 8 weeks of gestation used for the co-culture study. DS, decidual side; FV, floating villi. (E) Immunofluorescent assessment using the indicated antibodies on the serial sections from the co-cultured placental villi-decidua (*n*=6). TC, trophoblast cell column. Scale bars: 100 µm.

The huge difference in the expression level of PLAC8 between iEVTs and CTBs, the progenitors of EVTs, suggest that the expression profile of PLAC8 was precisely controlled during the differentiation of CTBs into iEVTs. To test this hypothesis, we first used the well-established iEVT induction model, in which CTBs that were isolated from 5- to 7-week-old placentas gradually become iEVTs during *in vitro* culture at 72 h, as illustrated by the emergence and the time-dependent increase in the expression of the EVT markers HLA-G and integrin α5 (Fig. 3A-C, Fig. S3). Meanwhile we observed that PLAC8 was barely present in the primary CTBs, but its levels were greatly increased during the differentiation process (Fig. 3A-C), suggesting that PLAC8 was induced at the beginning of the differentiation of CTBs and was induced more and more during this process in the *in vitro* culture system.

To mimic the *in vivo* differentiation process, we set up another iEVT differentiation model, where floating villi (without the existence of visible TCs) from 8-week-old human placentas were co-cultured with the corresponding maternal decidua block (Fig. 3D). During a co-culturing at 72 h, a TC was formed at the tip of a former villus that interacted with the decidua, and iEVTs originated from the induced TC migrated and invaded the decidua. An immunofluorescent staining assay showed that whereas PLAC8 was hardly detectable in the villous trophoblasts, both the TC area and the iEVTs were positive for PLAC8 (Fig. 3E). Taken together, these findings suggest that expression of PLAC8 was greatly induced during the differentiation process of CTBs into iEVTs.

### Oxygen tension regulates the expression of PLAC8 in human EVTs

**Fig. 4.**
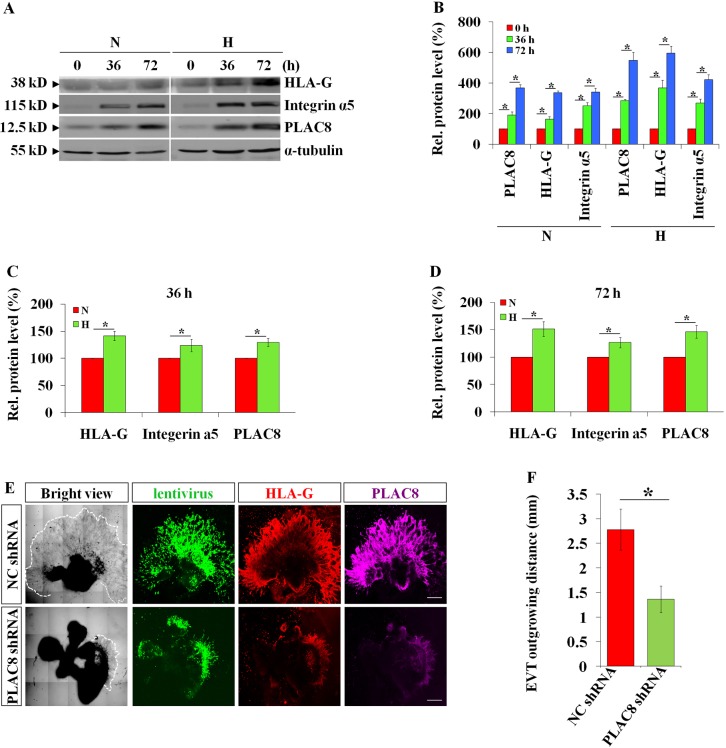
**Oxygen tension regulates the expression of PLAC8 in human EVTs.** (A) Western blotting of induced EVTs differentiated from first trimester CTBs under different oxygen tensions using the indicated antibodies. (B-D) Statistical analysis of A. N, normoxia (oxygen tension of 20%); H, hypoxia (oxygen tension of 2%). The expression levels of PLAC8, integrin α5 and HLA-G were significant higher for the induced EVTs under hypoxia (2% oxygen tension) environment than normoxia (20% oxygen tension) at 36 h (C) or 72 h (D) of treatment (*n*=5). *P<0.05. (E) Lentiviral-mediated PLAC8 knockdown obviously inhibited EVT differentiation in an *ex vivo* first trimester placental villi explant EVT induction model (visualized using immunofluorescence). Scale bars: 1 mm. (F) Statistical analysis of the outgrowth distance of EVTs, as representatively shown in E (*n*=16, *P<0.05).

How is PLAC8 regulated during human iEVTs differentiation? During the process of early placentation, the occlusion of the maternal spiral arteries by trophoblast cells generates low-oxygen conditions at the fetomaternal interface, which has been found to play a pivotal role in regulating trophoblastic lineage differentiation (Caniggia et al., 2000; Genbacev et al., 1997; Kaufmann and Castellucci, 1997). Noting that the expression level of PLAC8 is tightly controlled during trophoblast differentiation, we sought to determine whether PLAC8 expression correlates with oxygen tension conditions. To achieve this, we again used the *in vitro* iEVT induction model, where primary CTBs were cultured under normoxic (oxygen tension of 20%) or hypoxic (oxygen tension of 2%) (Genbacev et al., 1997) conditions. As shown in Fig. 4A-D, the PLAC8 expression level was significantly higher under hypoxia, compared with that in normoxia, suggesting an essential role for oxygen tension in controlling PLAC8 induction. In order to further verify whether the upregulation of PLAC8 expression is definitely oxygen tension dependent, we seeded cells from the extravillous trophoblast cell line HTR8/SVneo onto the matrigel to mimic iEVT differentiation (Fig. S4A), as indicated by the obvious increases in *HLA-G*, matrix metalloproteinase (*MMP*) *2* and *MMP9* mRNA levels (Fig. S4B). Low-oxygen condition increased the expression of hypoxia inducible factors (HIFs) 1α and 2α, as expected, and activated expression of PLAC8 (Fig. S4C,D). We further treated HTR8/SVneo cells with CoCl_2_ (to induce hypoxia; 200 mM) and FM19G11 (final concentration from 0.1 to 62.5 mM with a fivefold gradient increment), an inhibitor of HIFα, and found that prevention of HIF signaling via FM19G11 greatly inhibited the PLAC8 induction under hypoxic condition (Fig. S4E,F). Collectively, our data suggest that PLAC8 induction during iEVTs differentiation is oxygen tension dependent.

### PLAC8 is essential for differentiation of CTBs into iEVTs

Our observations that cell differentiation of CTBs into iEVTs during placental development correlates with precise upregulation of PLAC8 protein level and previous findings that mouse PLAC8 regulates the differentiation of acute myeloid leukemia cells (Wu et al., 2010) naturally made us wonder whether this newly gathered PLAC8 might contribute to the CTBs differentiation process. To test this hypothesis, we used another *ex vivo* placental villi explant culture model, where human placental explants from the first trimester were cultured on matrigel and treated with lentivirus carrying either control shRNA or *PLAC8* shRNA. After 96 h of culture, we harvested the explant together with the outgrowing iEVTs attached on the matrigel, and performed whole-mount immunofluorescence assays. As shown in Fig. 4E, the cells that migrated out of the villi in the control group (Fig. 4E, upper panel) were positive for both HLA-G and PLAC8, indicating that they are differentiated iEVTs. However, placental explants that were pretreated with *PLAC8* shRNA (Fig. 4E, lower panel) displayed not only very low protein levels of PLAC8, but also a much smaller amount of iEVTs (Fig. 4F), suggesting iEVTs differentiation was significantly suppressed when PLAC8 was abolished. Collectively, our data indicate that PLAC8 is necessary for the differentiation of CTBs into iEVTs.

### PLAC8 promotes cell invasion and migration

As PLAC8 is highly abundant in iEVTs and the major function of iEVTs is migrating and invading into the maternal decidua, we then hypothesized that PLAC8 might play an important role during these processes. To test this hypothesis, a stable EGFP-tagged PLAC8-expressing HTR8/SVneo cell line (HTR8/SVneo-PLAC8-EGFP) was established (Fig. S5A,B). We then performed matrigel cell invasion and transwell cell migration assays and found that the HTR8/SVneo-PLAC8-EGFP cells displayed much higher capacities of invading and migrating than those in the control cells, which were transfected with the empty vector (EV) (Fig. S5C,D). Further wound-healing assays confirmed the conclusion that increased PLAC8 levels positively contributed to cell migration capacity (Fig. S5E,F). To further detect whether the invasion and migration of HTR8/SVneo cells was dependent on the existence of PLAC8, we first performed rescue experiments by again introducing the *PLAC8* siRNA1 and siRNA2 into HTR8/SVneo-PLAC8-EGFP and control cells (Fig. S6). As shown in Fig. S5G,H, knockdown of PLAC8 dramatically attenuated the enhanced cell migration abilities by overexpression of PLAC8-EGFP via cell wound-healing assays.

**Fig. 5.**
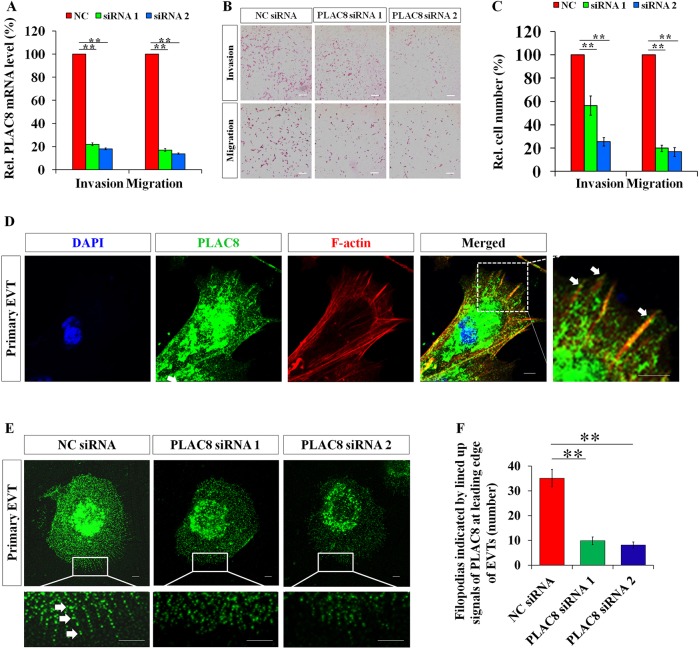
**PLAC8 promotes cell invasion and migration.** (A-C) Analysis of the invasive and migratory potential of the induced EVTs differentiated from primary CTBs and the statistical analysis (C, *n*=5, **P<0.01). (A) The knockdown efficiency on *PLAC8* mRNA using indicated siRNAs. (B) Representative images of transwell membranes containing invaded or migratory cells under indicated treatments. Scale bars: 200 µm. (C) Statistical analyses on invasion and migration assays as representatively shown in B (*n*=5; **P<0.01). (D) Immunofluorescence of extravillous trophoblast cells using indicated antibodies showing the partial colocalization of PLAC8 with the stress fiber at the cell leading edge (white arrows) in an explant culture model. (E) Representative immunofluorescent images of filopodia in the primary human trophoblast cells with indicated treatments. Arrows indicate filopodia. (F) A statistical assay showing the decrease in the number of filopodia after PLAC8 siRNA treatment, as representatively shown in E (*n*=12; **P<0.01). Scale bars: 5 µm in D,E.

Next, we repeated the above experiments in the primary trophoblast cells. *PLAC8* siRNA1 and siRNA2 were introduced 24 h before trypsinization of the cultured trophoblast cells and the cells were then applied to the matrigel cell invasion and transwell cell migration assays. Real-time qPCR showed *PLAC8* mRNA knockdown efficiency of more than 80% by siRNA1 and siRNA2 (Fig. 5A), and the invasion and migration of the primary EVT cells were significantly inhibited by these siRNAs (Fig. 5B,C). Taken together, our data strongly suggest that PLAC8 positively regulates invasion and migration of iEVTs during placental development.

### PLAC8 localizes at leading edge and regulates the activity of small GTPase

In order to investigate the regulatory mechanism whereby PLAC8 promotes invasion and migration of EVTs, we first analyzed the cytoplasmic distribution of PLAC8. Immunofluorescence staining of primary EVTs showed that PLAC8 localized at cytoplasm as punctate signals around the nucleus, as previously reported (Li et al., 2014; Rogulski et al., 2005), and was also detected as dot-like signals lined up along the actin filaments at the leading edge (Fig. 5D-F) of the migratory EVTs from a trophoblast explant culture system, suggesting PLAC8 localized at the cell periphery.

**Fig. 6.**
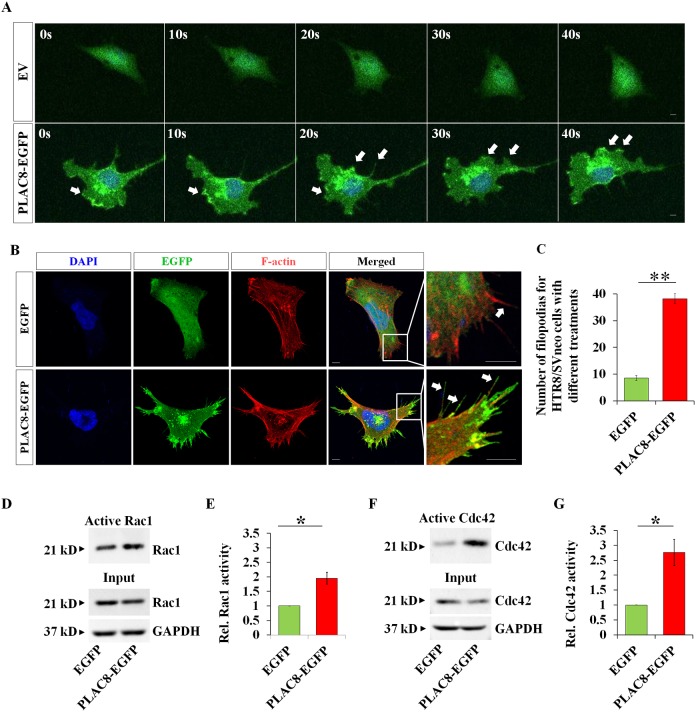
**PLAC8 increases the activation of Rac1 and Cdc42, and determines the number of filopodias of migratory cells.** (A) Still images of a time-lapse imaging (Movie 1) showing the localization of PLAC8-EGFP (arrows) during the migration of HTR/SVneo cells. EV, empty vector. (B) Representative immunofluorescent images of filopodia (arrows) in HTR8/SVneo cells stably transfected with empty vector (EV, upper panels) or PLAC8-EGFP (lower panels). (C) A statistical assay showing the increase in the number of filopodia after PLAC8 overexpression, as representatively shown in B (*n*=12; **P<0.01). (D-G) Western blots and statistical analyses showing the activation of Rac1 (D,E) and Cdc42 (F,G) after overexpression of PLAC8 in HTR8/SVneo cells (*n*=5, *P<0.05). GAPDH is a loading control. Scale bars: 5 µm.

To further examine the biological function of PLAC8, we performed time-lapse recording using HTR8/SVneo cells stably expressing EGFP or PLAC8-EGFP. Compared with control cells, the migrating PLAC8-EGFP expressed cells had more filopodia- and lamellipodium-like structures at the leading edge, and these protrusions were very dynamic (Fig. 6A, Movie 1), suggesting that PLAC8 may be involved in the formation of filopodia and lamellipodia. To test this idea, we performed a spreading assay and subsequently immunostaining for F-actin and GFP using the same cell lines. The PLAC8-EGFP stably expressed HTR8/SVneo cells exhibited much more filopodias compared with control cells (Fig. 6B,C). Remarkably, PLAC8-EGFP signals significantly accumulated at tips of filopodia (Fig. 6B), consistent with distributions of endogenous PLAC8 (Fig. 5D). Taken together, these observations strongly suggest expression PLAC8 promotes the dynamics of leading-edge protrusions.

As the filopodia and lamellipodia formation was dynamically regulated by the activities of small GTPases, such as Cdc42 and Rac1, we looked at the activities of Cdc42 and Rac1with or without PLAC8 expression. Results revealed increased activity of Cdc42 and Rac1 in cells expressing PLAC8-EGFP (Fig. 6D-G). In addition, no significant changes in protein expression or post-translational levels of Cdc42 and Rac1 were observed with western blots or using RNA-seq methods (Fig. 6D-G, Table S4). These results suggest that the regulation of PLAC8 on protrusions at cell periphery might be achieved by modulating the activity of the small GTPases Cdc42 and Rac1.

### The PLAC8 levels in the iEVTs from patients with severe PE are higher when compared with their gestational stage-matched controls

**Fig. 7.**
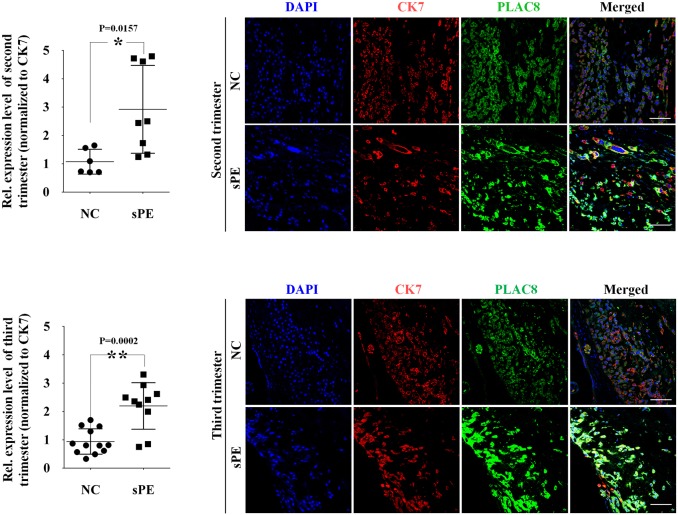
**iEVTs from sPE patients display increased level of PLAC8.** Immunofluorescent assessment of PLAC8 expression in iEVTs from the second (top) and third (bottom) trimester sPE placentas (*n*=18), and the gestational stage-matched normal controls (*n*=18) (right panels) and quantifications (left panels). The signals were collected under the same confocal scanning parameters and were normalized to CK7 before being evaluated simultaneously using the same software: ZEN 2011. NC, normal control; sPE, severe preeclampsia. Scale bars: 200 µm. **P<0.01, *P<0.05.

Shallow invasion of iEVTs into the maternal decidua and poor remodeling of the maternal spiral arteries have been widely recognized as the primary cause of PE onset (Kam et al., 1999; Kaufmann and Castellucci, 1997). Placental villi from severe PE (sPE) patients (Table S1A,B, second trimester and third trimester, respectively) and normal pregnant women at matched gestational stages were collected and sectioned to immunolocalize PLAC8. In all of the sPE placental paraffin sections from the second (Fig. 7A) and the third (Fig. 7B) trimesters, PLAC8 was also specifically localized in iEVTs using immunofluorescence (Fig. 7A,B, right panels). After normalizing to the level of CK7, an internal control, in the same population of cells and after the statistical analysis, the levels of PLAC8 were found to be higher in the iEVTs from the sPE placentas when compared with the trimester matched normal control placentas (Fig. 7A,B, left panels).

## DISCUSSION

It is very valuable to comprehensively define the specific molecular markers for trophoblast cells, particularly their subpopulations in the human placenta, which would be fundamental to understand their specific characteristics and functions. In the present study, we show that PLAC8 is a novel membrane-localized iEVT marker, based on the following evidence: (1) PLAC8 is exclusively expressed in iEVTs at both the transcript and protein levels throughout pregnancy; (2) in three primary iEVT-induction models, including differentiation of CTB into iEVTs, co-culture of the placental floating villi and decidua, as well as the *in vitro* trophoblast explant culture model, PLAC8 expression is intensively induced in the newly developed iEVT; (3) PLAC8 is absent in eEVTs and other trophoblast cell types, including cytotrophoblast cells and syncytiotrophoblast. To our knowledge, PLAC8 is the first molecular marker that can be used to distinguish iEVTs from eEVTs.

### The difference in PLAC8 levels between iEVTs and eEVTs could suggest separate differentiation routes of these two populations of EVTs

Regarding eEVT lineage differentiation, one hypothetical theory is that the iaEVTs plug in the spiral artery at the first trimester from the EVT shell, and they further develop into the imEVTs (Harris et al., 2009; Pijnenborg et al., 2006). The second theory is that eEVTs originate from a process called ‘intravasation’, the inward movement of iEVTs into the vessels from the outside and the subsequent differentiation to form the eEVTs (Kaufmann and Castellucci, 1997). During early pregnancy, the spiral arteries become surrounded by iEVTs, resulting in a marked perivascular clustering of iEVTs in the superficial decidua (Pijnenborg et al., 1980). Therefore, it is highly possible that in most superficial decidua, at least some of the eEVTs are derived from the numerous iEVTs via intravasation. If this is true, we would expect a gradual increase or decrease in the expression of certain genes from the extra-arterial iEVTs to inter-arterial imEVTs to intra-arterial iaEVTs. However, the ‘yes or no’ expression pattern of PLAC8 in iEVTs and eEVTs, respectively, found in this study suggests that eEVTs may not be derived from iEVTs that inhabit in decidua.

### Oxygen tension-dependent expression of PLAC8 regulates the differentiation of EVTs and may correlate to the onset of preeclampsia

Oxygen tension is important for trophoblast cell differentiation and invasiveness (Caniggia et al., 2000; Genbacev et al., 1997). Low oxygen tension can enhance trophoblastic invasion by upregulating the c-met proto-oncogene product, which is activated by HIF1α (Hayashi et al., 2005). However, the possible role of gradients of oxygen tension in directed EVT invasion and migration has not been well elucidated. In this study, we observed significant elevations in PLAC8 expression in response to hypoxia in both primary induced iEVTs and the EVT cell line, and the increase in PLAC8 in HTR8/SVneo cells is attenuated by inhibiting the hypoxic conditions. These results suggest that PLAC8 may serve as a regulatory sensor of oxygen tension to direct differentiation of CTBs into iEVT during pregnancy. The finding that PLAC8 is exclusively expressed in the interstitial EVTs instead of eEVTs further supports this notion as the interstitial spaces of the decidua have a relatively low oxygen tension environment compared with the uterine spiral arteries. Whether PLAC8 is a key molecule that can sense the oxygen tension to control iEVT and eEVT differentiation remains to be further investigated.

A typical pathological characteristic that arises from the poorly perfused placenta of preeclampsia patients is a relatively more hypoxic status at the fetomaternal interface (Mise et al., 1998; Soleymanlou et al., 2005). Expectantly, we observed that PLAC8 was highly abundant in the preeclamptic placentas. However, the current widely accepted etiology of preeclampsia, defects in EVT differentiation and the subsequent shallow invasion of iEVTs in the decidua (Pijnenborg et al., 2006; Zhou et al., 1993), would predict a decrease in the expression of PLAC8, which is opposite to the results found in this study. We speculate that the elevated expression of PLAC8 could have compensatory roles to relieve the adverse pathogenic progress attributed to the restricted iEVT invasion. A second interpretation is that PLAC8 expression might have a physiological range for invasion and motility, where too high or too low both causes a decrease in invasion and motility. A third possibility is that PLAC8 may be essential to cell fate of EVTs to balance the ratios of EVT cell types. The increase in PLAC8 in sPE may cause more iEVT cells and fewer eEVT cells, leading to the observed failure of spiral artery remodeling. Further studies are required to address the issues of how PLAC8-associated network contributes to the differentiation of CTBs into iEVTs, so as to ensure appropriate development of a functional placenta.

### PLAC8 enhances trophoblast invasion and migration partially through regulating Rho GTPase activity

In this study, we found that PLAC8 promotes invasion and migration of human trophoblast cells, which further our understanding on the already very complicated mechanisms of human trophoblast invasion and migration (Bischof et al., 2002; Ferretti et al., 2007; Huppertz et al., 1998; Kaufmann and Castellucci, 1997; Lala and Hamilton, 1996; Pollheimer and Knöfler, 2005).

To achieve directed cell migration, cells should sequentially undergo the process of sensing the chemotactic stimulation, establishing polarity of subcellular structures, forming protrusions and adhering in the direction of migration, followed by retraction and translocation under the driving force supplied by actin dynamics (Bravo-Cordero et al., 2012; Ponti et al., 2004). We subsequently found that PLAC8 is localized on the cell leading edge as dots-like signals, which lined up along actin filaments. More interestingly, PLAC8 could enhance the activation of Rac1 and Cdc42, which further leads to a stronger ability to form more filopodias and lamellipodium at the leading edge of a polarized trophoblast cell. Thus, we believe that PLAC8 is a major player in the formation of protrusions and filopodias, thus promoting invasion and migration of trophoblast cells.

In summary, we have identified PLAC8 as a novel marker for iEVT. The abundant expression of PLAC8 in iEVT could positively regulate trophoblast invasion and migration partially through upregulating the activation of Rac1 and Cdc42.

## MATERIALS AND METHODS

### Informed consent and sample collection

This research was approved by the Ethics Committee of the 306th Hospital of PLA (Beijing, China). Signed informed consent was obtained from all women who donated their placentas. Samples were used according to standard experimental protocols that were approved by the Ethics Committee of the Institute of Zoology, Chinese Academy of Sciences. First trimester placental villi and the corresponding decidua samples were obtained from women undergoing elective surgical termination of their pregnancies between 5 and 8 weeks of gestation. Placental tissues from second and third trimesters were collected from women undergoing Cesarean sections. The mid-gestation placentas and the pre-term birth placentas employed as normal controls of PE were obtained from women with cervical incompetency without infection or other possible diseases that would increase the risk of abortion or affecting placentation. PE was defined as a new onset of hypertension (systolic/diastolic blood pressure ≥140/90 mm Hg measured on two occasions at least 4 h apart) and proteinuria (≥300 mg per 24 h) after 20 weeks of gestation (those with a systolic/diastolic blood pressure ≥160/110 mmHg and proteinuria ≥5000 mg per 24 h were deemed as sPE). None of the PE patients involved in the current study presented with complications.

### Immunofluorescence and immunohistochemistry

The procedure for immunofluorescence and immunohistochemistry and setting of controls were exactly as previously reported (Chang et al., 2014) and are detailed in the supplementary Materials and Methods. The information and rules of use of primary antibodies are in Table S2. Combinations of primary antibodies for dual immunofluorescence staining are in Table S3.

### *In situ* hybridization

The procedures for *in situ* hybridization and setting of controls were as previously reported and detailed in the supplementary Materials and Methods.

### Isolation of cytotrophoblast cells and *in vitro* EVT induction

The placental villi (5-7 weeks of gestation, 20 placentas each time, *n*=5) were minced and digested with 0.25% trypsin and 0.02% DNase type I (Sigma-Aldrich) four times at 37°C to detach the CTBs. The cell suspension from the first digestion was discarded, and those from the following three digestions were carefully loaded onto a Percoll gradient (70% to 10%, in 10% steps), then centrifuged at 1200 ***g*** for 25 min. Cells that sedimented at the 30%-40% region of the Percoll gradient were collected (94.6% were CTBs). To differentiate CTBs into EVTs, CTBs were seeded onto a six-well plate coated with liquid matrigel (1 μg/μl; BD Biosciences) at 2.5×10^6^ cells per well and cultured in DMEM/F12 supplemented with 10% fetal bovine serum for 48 or 72 h. After 48 h of culture, 96% of the cells were induced EVTs.

### Co-culture of placental villi and decidua from the first trimester

Floating villi at 6-10 weeks of gestation were dissected under a phase-contrast microscope (Leica S6 D Stereozoom). Small pieces (0.5×0.5 cm) of decidua with no anchored villi from the same individual were also collected and mounted onto transwell inserts (Millicell Cell Culture Inserts, 0.4 µm, 12 mm diameter, PICM01250, Merck Millipore) containing matrigel (5 mg/ml). Counterparts from the same decidua were used for immunofluorescence to examine whether trophoblast cells are present or not. After the decidua was well mounted on the matrigel, three to eight floating villous trees without columns were explanted to the upper surface of the mounted decidua. The co-cultured tissues were fixed with 4% PFA after being cultured for 3 days.

### Human extravillous explant culture and lentiviral infection

Human first trimester placental villi (5-6 w, *n*=16) were collected and anchoring villi with attached trophoblast cell columns were selected and dissected into explants with diameters of 2 to 5 mm. The explants were implanted onto Millicell-CM culture dish inserts (Millipore Corporation, Bedford, MA) coated with growth factor-reduced matrigel (5 μg/μl) and cultured for 8 h. Lentiviruses carrying *PLAC8* shRNA (targeting to 5′-GGUGGUCGUUGUGACCCAACCUGGA-3′) or the universal control shRNA (hU6-MCS-CMV-EGFP lentivirus system; Genechem, Shanghai, China) were added into the upper chamber for 16 h. Then, serum-free DMEM/F12 was added into the culture dish insert chamber and 500 μl of DMEM/F12 (supplemented with 10% FBS, 100 units/ml penicillin, 100 μg/ml streptomycin and 2.5 μg/ml fungizone) was added to the lower well. The infection efficiency could be monitored based on the expression of green fluorescent protein. Twenty-four hours after a successful infection, the explants were observed and recorded using an inverted microscope system (Nikon Eclipse Ti). Only the explants with successful initiation of EVT outgrowth from the trophoblast cell column were selected. Explants from the same placental villi were paired as experimental (*PLAC8* shRNA-treated) and control (universal negative control shRNA-treated) groups.

### Cell line, RNA interference and PLAC8 overexpression

HTR8/SVneo cells used in this study were a gift from Dr Benjamin K. Tsang (Chronic Disease Program, Ottawa Health Research Institute, Ottawa, ON, Canada). Culture of the cells and detailed procedure for RNAi and overexpression are in the supplementary Materials and Methods. *PLAC8* siRNA had the sequences 5′-GGUGGUCGUUGUGACCCAACCUGGA-3′ and 5′-CAAAUCAAGAGAGAUAUCAACAGAA-3′ (Invitrogen). The GenBank accession number for PLAC8 is NM_001130716.1.

### Matrigel invasion and transwell migration assays

The experiments were performed as previously reported (Chang et al., 2014) and are detailed in the supplementary Materials and Methods.

### Wound-healing assay

The experiments were performed as previously reported (Liang et al., 2007) and are detailed in the supplementary Materials and Methods.

### Live-cell imaging

An Eclipse Ti model inverted microscope with a 20× objective lens (numerical aperture, 0.95; Nikon) and an Orca ER model camera (Hamamatsu) was used. The filter sets used in this assay were: Hoechst 33342 (405 nm) and PLAC8-EGFP (488 nm). The time lapse images were recorded at 10 min intervals for 48 h. Image acquisition was controlled using Volocity software (Perkin-Elmer).

### RNA sequencing

A detailed protocol of the RNA sequencing experiments is provided in the supplementary Materials and Methods.

### Rac1 and Cdc42 activity assay

Subcloned PGEX-4T1 bacterial expression vector with GST bound human PAK1 binding domain (GST-PAK1-PBD) was expressed in *E. coli* strain *DH5a* and purified with Glutathione Sepharose 4B beads as instructed by the manufacturers. Cells were lysed with ice-cold lysis buffer [50 mM Tris (pH 7.5), 10 mM MgCl_2_, 500 mM NaCl, 1% Triton X-100] supplemented with protease inhibitor cocktail and phenylmethylsulfonyl fluoride (PMSF) and cleared by centrifugation at 12,000 ***g*** for 5 min at 4°C. Purified GST-PAK1-PBD proteins and Glutathione-Sepharose 4B beads were added to the cell lysates and incubated at 4°C for 30 min with rotation to precipitate activated Rac1 and Cdc42. The beads were washed and boiled with sodium dodecyl sulfate (SDS) protein loading buffer and subjected to western blot detection using anti-Rac1 (610651, BD Biosciences, 1:3000) and anti-Cdc42 (610929, BD Biosciences, 1:1000) antibodies.

### Statistical analysis

The data were analyzed using one-way ANOVA or paired *t*-tests using the Statistical Package for the Social Sciences (SPSS for Windows package release 10.0). The results are expressed as the mean±s.e.m. *P*<0.05 was considered significant.

## Supplementary Material

Supplementary information
