# Correction: PLAC8, a new marker for human interstitial extravillous trophoblast cells, promotes their invasion and migration

**DOI:** 10.1242/dev.204982

**Published:** 2025-08-15

**Authors:** Wen-Lin Chang, Ya-Wei Liu, Yan-Li Dang, Xiang-Xiang Jiang, Honglin Xu, Xing Huang, Yan-Ling Wang, Haibin Wang, Cheng Zhu, Li-Qun Xue, Hai-Yan Lin, Wenxiang Meng, Hongmei Wang

There was an error in *Development* (2018) **145**, dev148932 (doi:10.1242/dev.148932).

The authors contacted us to say that they had inadvertently used the same immunofluorescence assay of HLA-G in two separate figure panels, Fig. 3C and Fig. S3.

**Fig. 3:**
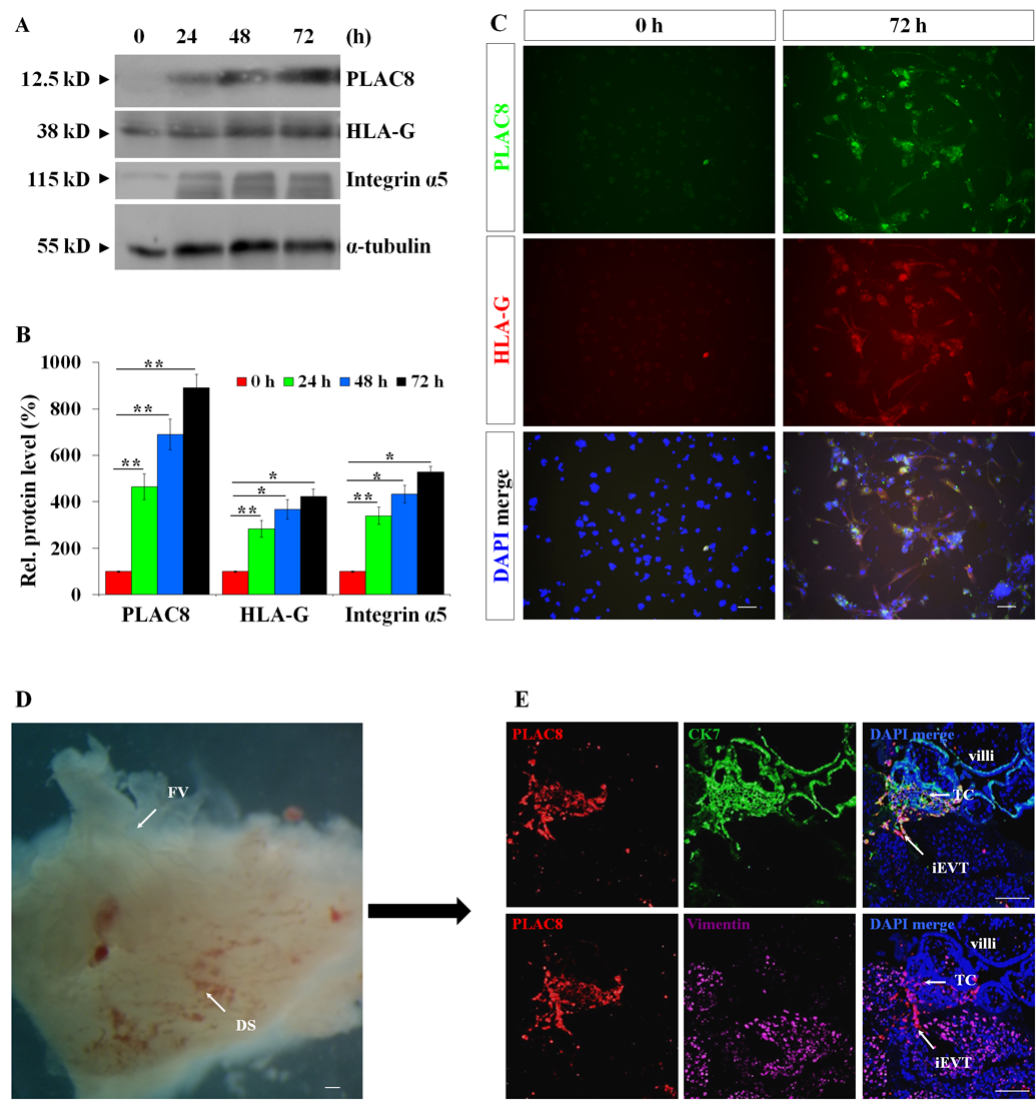
**(corrected). PLAC8 expression is induced during the differentiation of CTBs into iEVTs.** (A) Successful iEVT differentiation from CTBs is indicated by the increased expression levels of the EVT markers HLA-G and integrin α5 by western blotting. α-Tubulin is a loading control. (B) Statistical analysis of the western blotting results representatively shown in A (*n*=5; *P<0.05, **P<0.01). (C) Immunofluorescent assessment of CTB cells (0 h and 72 h) using the indicated antibodies in the *ex vivo* EVT induction model. (D) Morphology of placental villi and decidua from 8 weeks of gestation used for the co-culture study. DS, decidual side; FV, floating villi. (E) Immunofluorescent assessment using the indicated antibodies on the serial sections from the co-cultured placental villi-decidua (*n*=6). TC, trophoblast cell column. Scale bars: 100 µm.

A replacement panel from the original data has been substituted. The corrected Fig. 3 is shown below.

The authors apologise for this error, which does not impact the results and conclusions of the paper.

