# Supplementary material for: PLAC8, a new marker for human interstitial extravillous trophoblast cells, promotes their invasion and migration
Source: Development. 2018 Jan 15;145(2):dev148932. doi: 10.1242/dev.148932 (PMC5825838; doi:10.1242/dev.148932)
Supplement: Supplementary information [file develop-145-148932-s1.pdf]

Supplementary Figures

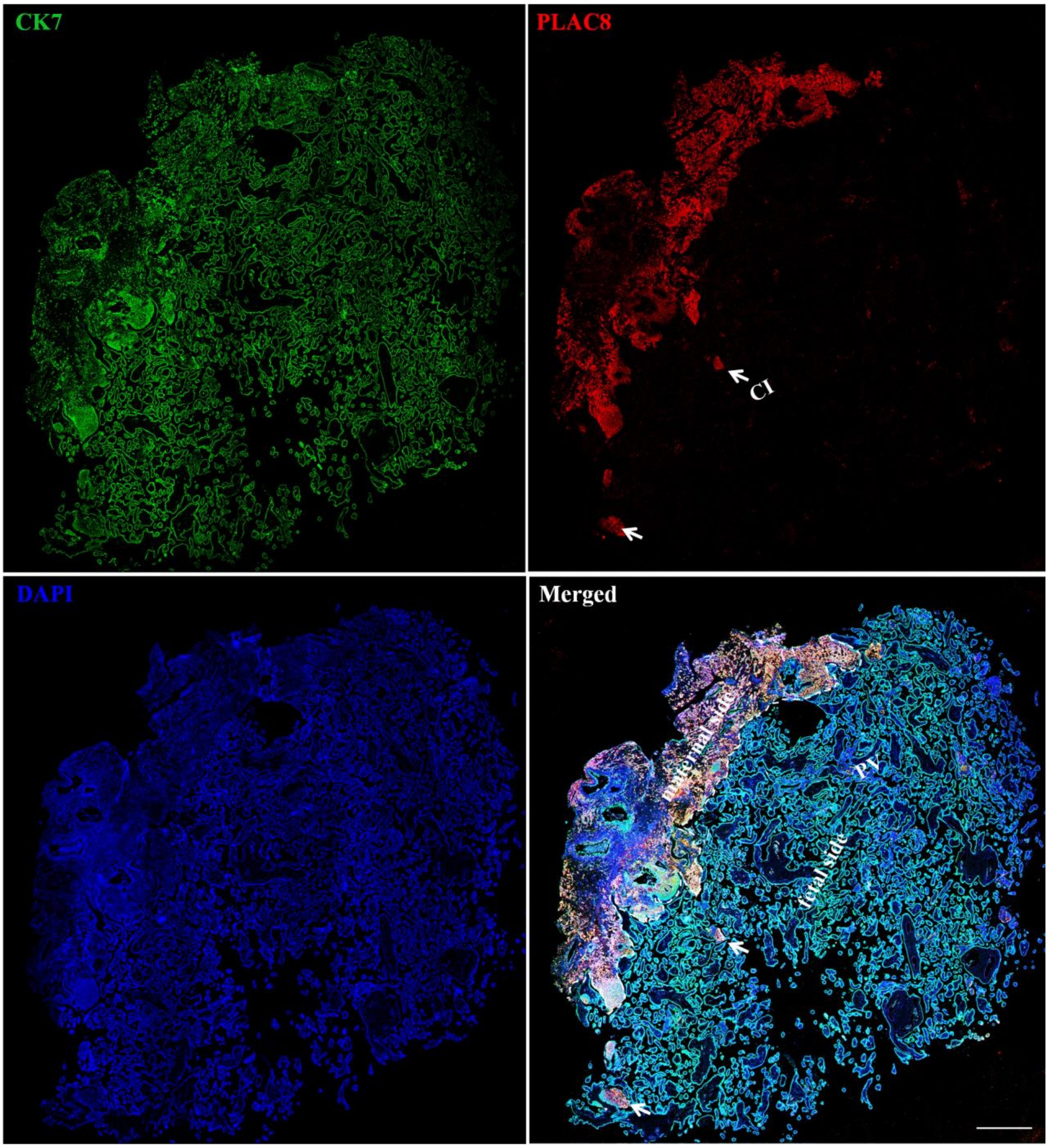

Fig. S1. A tile scan overview of PLAC8 expression pattern at the fetomaternal interface of the second trimester pregnancy (19 w). Placental villi (PV), cell islands (CI, white arrows) at the fetal side and EVTs at the maternal side were indicated by positive staining of CK7 antibody (green). PLAC8 (red) was exclusively expressed by iEVTs at the maternal side as well as the cell islands at the fetal side. DAPI is used to show nuclei. Scale bar, 1000 μm.

Development • Supplementary information

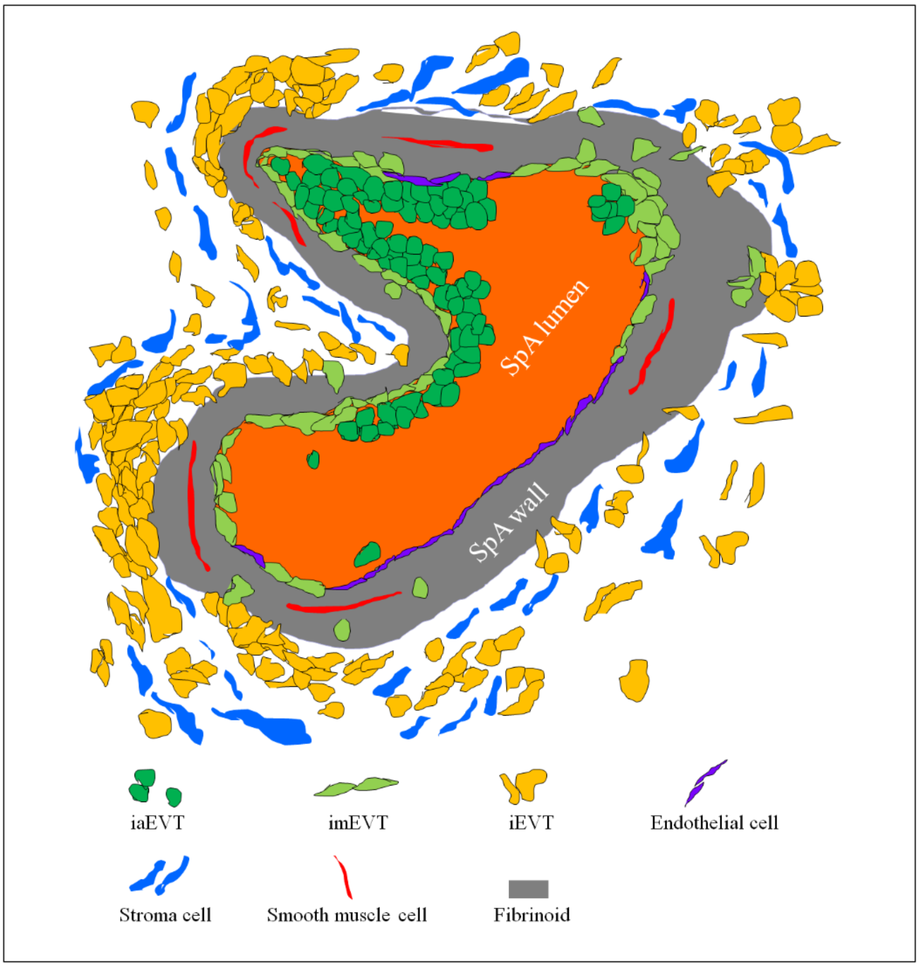

Fig. S2. A schematic graph showing the remodeling of a maternal spiral artery by trophoblast cells. iaEVTs, intra-artery extravillous trophoblast cells. imEVTs, intra-mural endovascular trophoblast cells. iEVT, interstitial EVT. SpA, spiral artery.

Development • Supplementary information

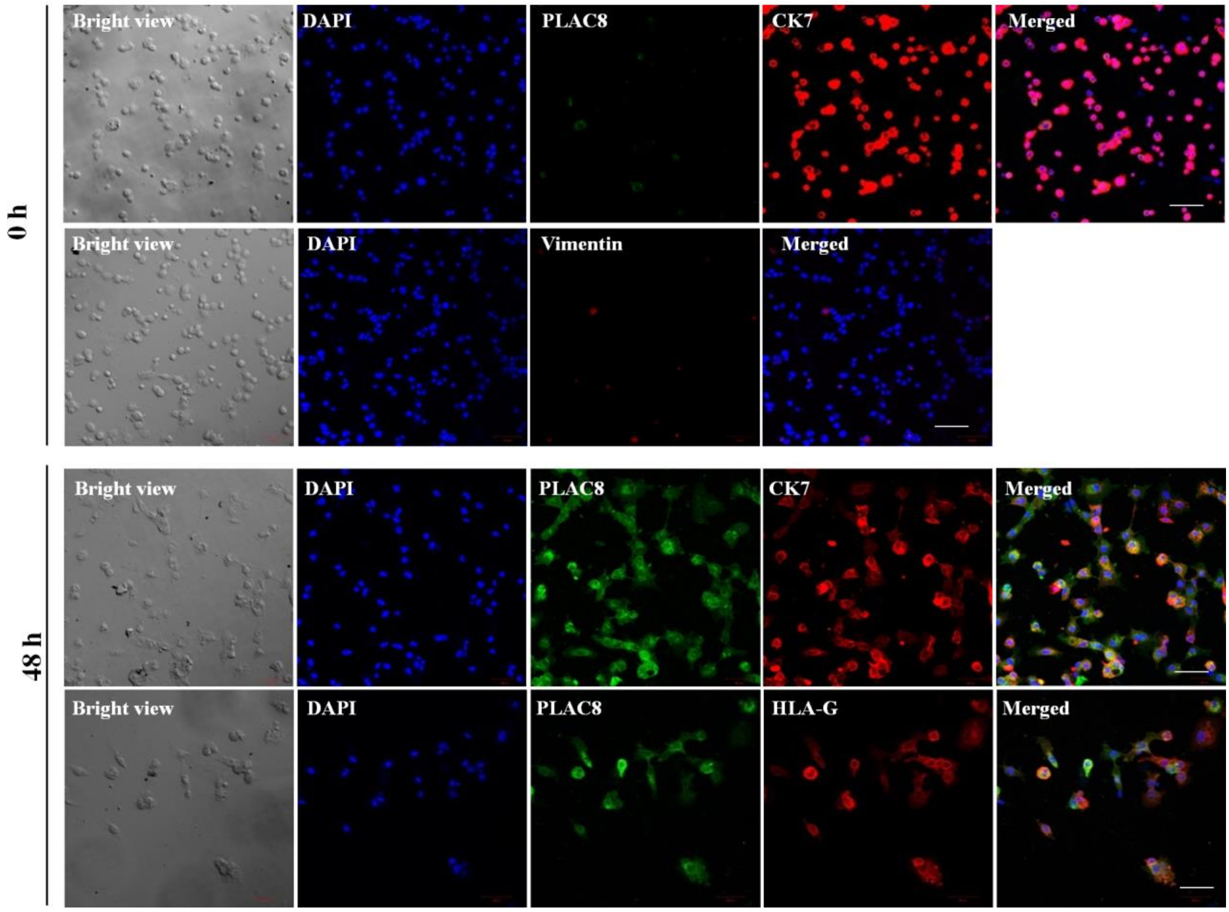

Fig. S3. IF assessment of CTB cells (0 h) and the induced iEVTs (48 h) using indicated antibodies in the *ex vivo* EVT induction model. The trophoblast characteristic of isolated CTBs (0 h) or induced EVT(s) was checked by incubation with anti-CK7 antibody. The purity of isolated CTBs was determined by incubation with anti-PLAC8 antibody (positive PLAC8 signal at time 0 h represented the EVTs from the remaining TC) and anti-Vimentin antibody (positive Vimentin staining represented the stroma cells). The inducing efficiency of CTBs to EVTs was measured by incubation with anti-HLA-G antibody at the time point of 48 h. The induced expression PLAC8 was affirmed by incubation with anti-PLAC8 antibody at the time point of 48 h. CTBs, cytotrophoblast cells. EVTs, extravillous trophoblast cells. TC, trophoblast column. Scale bar, 200 µm.

Development • Supplementary information

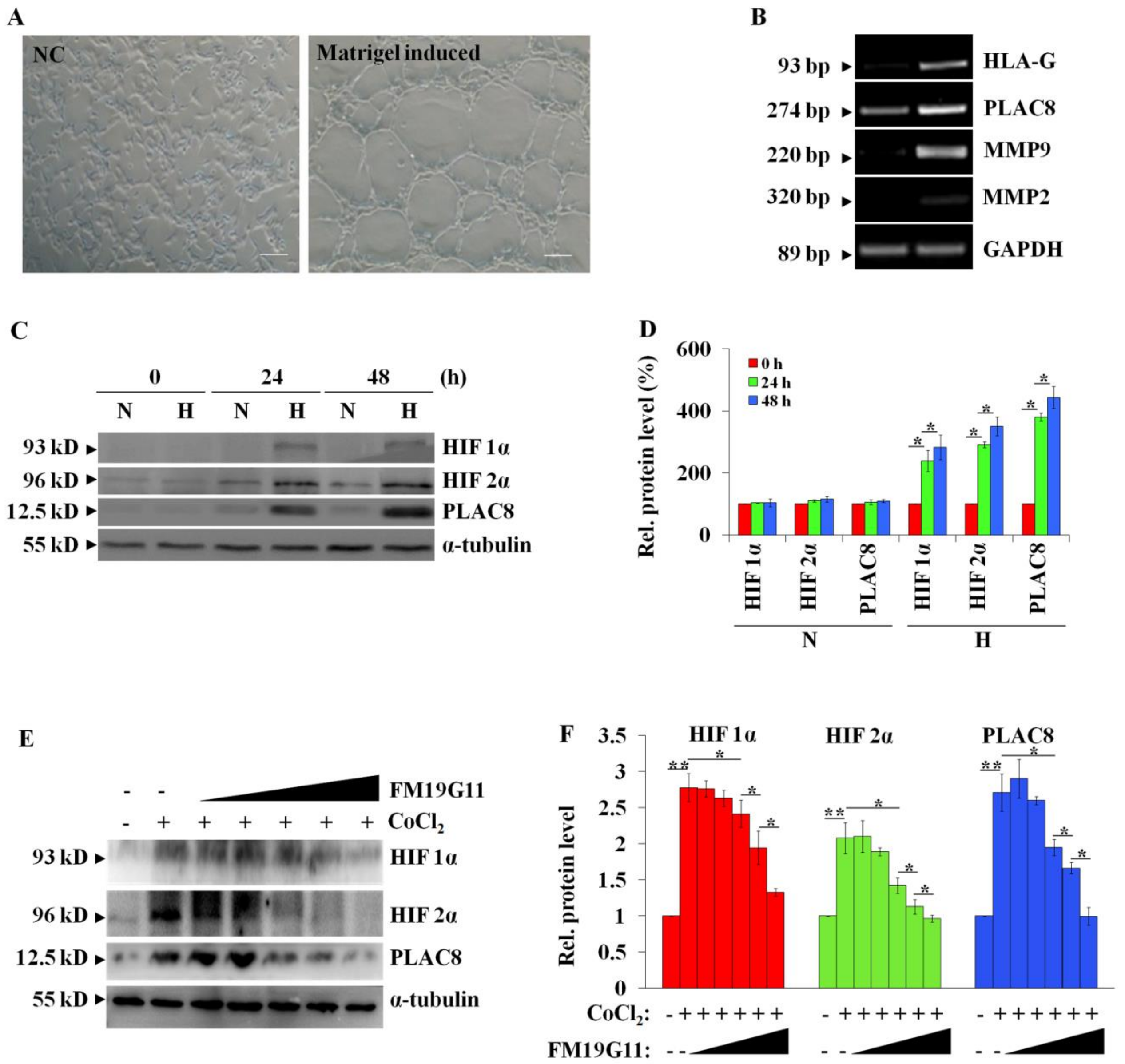

Fig. S4. Induction of tube formation in HTR8/SVneo cells and PLAC8 expression under hypoxia. (A) Tube formation was induced in HTR8/SVneo cells by matrigel (n=3). Scale bar, 200 µm. (B) Semi-quantitative RT-PCR assays showing the mRNA levels of indicated genes (n=3). GAPDH is an internal control. (C-D) Western blotting of HTR8/SVneo cells with the indicated treatment using indicated antibodies (C) and the statistical analysis (D, n=5; *, P<0.05). (E-F) Western blotting of HTR8/SVneo cells with indicated treatments using indicated antibodies (E) and the statistical analysis (F, n=5; *, P<0.05, **, P<0.01).

Development • Supplementary information

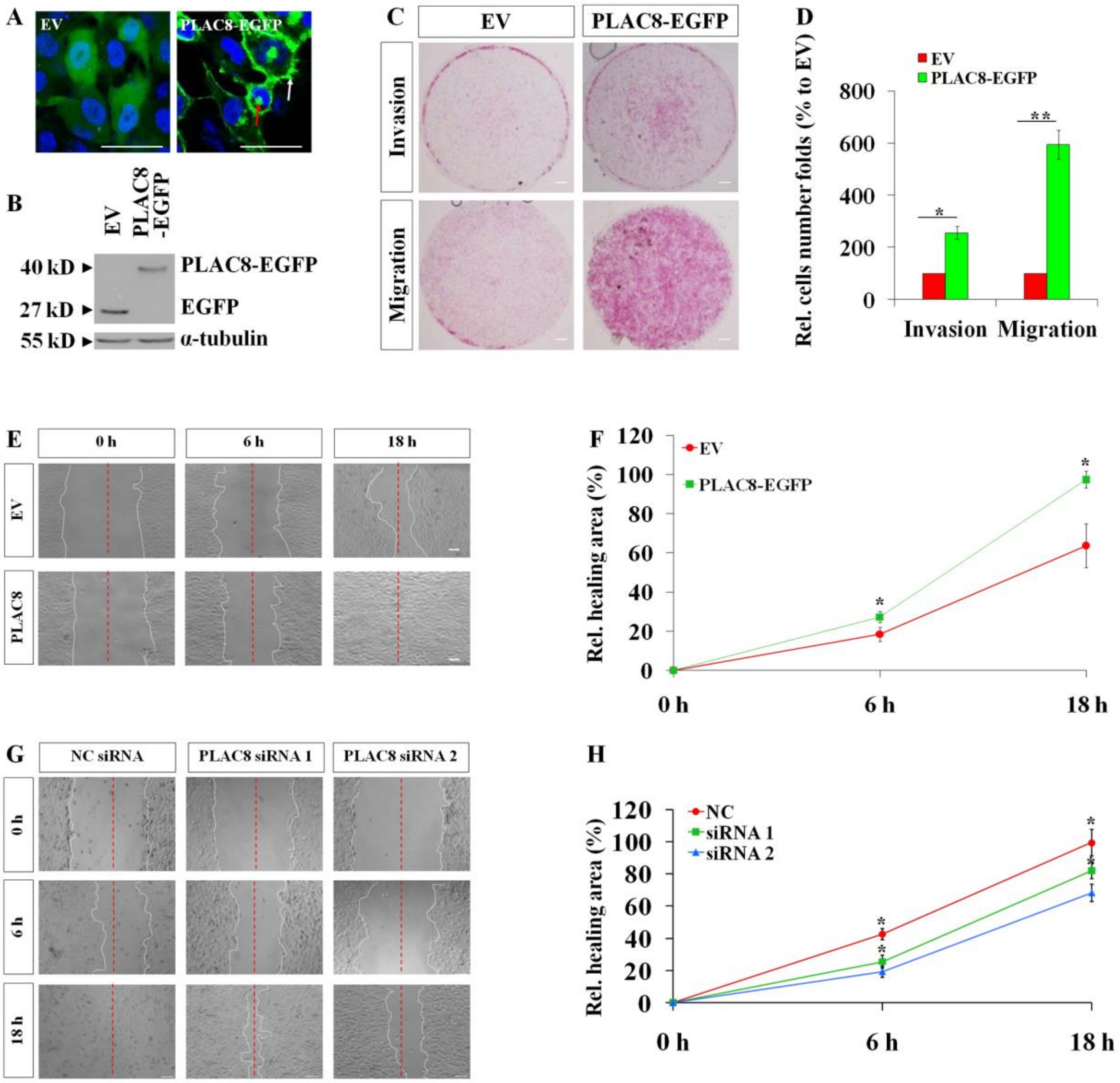

Fig. S5. PLAC8 promoted invasion and migration of HTR8/SVneo cells. (A) HTR8/SVneo cells were stably transfected with the empty vector pIRES2-EGFP (left) or pIRES2-PLAC8-EGFP (right). White arrowhead, filopodia; red arrowhead, localization adjacent to the nuclei. Scale bar, 50 μm. (B) Western blotting showing the overexpression of EGFP and the fused PLAC8-EGFP proteins. (C) Representative images of transwell membranes containing invaded or migratory cells that were stably transfected with EV or PLAC8-EGFP. Scale bar, 2000 μm. (D) Statistical analyses on invasion and migration assays as representatively shown in C (n=5; *, *P*<0.05, **, *P*<0.01). (E-F) The wound-healing assay on HTR8/SVneo-PLAC8-EGFP (PLAC8) or the control (EV) cells (E) and the statistical analysis (F, n=5; *, *P*<0.05). Scale bar, 200 μm. (G-H) Wound-healing assay on HTR8/SVneo-PLAC8-EGFP cells with indicated treatments (G) and the statistical analysis (H, n=5; *, *P*<0.05). Scale bar, 200 μm.

Development • Supplementary information

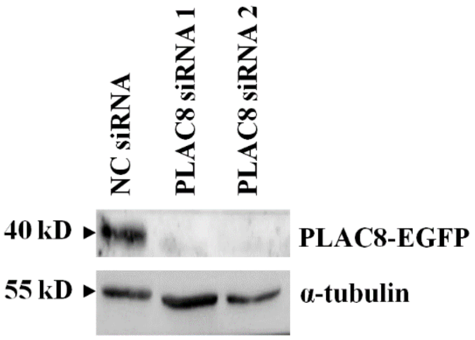

Fig. S6. PLAC8 knockdown efficiency in HTR8/Svneo-PLAC8-EGFP cells by indicated siRNAs using Western blot analysis. α-tubulin is a loading control.

Development • Supplementary information

Supplementary Tables

Table S1 a. Demographic and clinical characteristics of the normal pregnant women and sPE patients of second trimester enrolled in this study #.

Characteristics	Control (n =6)	sPE (n = 8)	*P* value
Maternal age (years)	28.34 ± 4.38	29.25 ± 3.21	NS
Preeclampsia onset (weeks)	None	20.56 ± 2.23	-
Gestational age at cesarean section (weeks)	24.125 ± 3.26	27.33 ± 2.25	$P<0.05^{*}$
Primiparae (%)	93.46%	86.32%	NS
Birth weight (g)	-	-	-
Female fetus (n)	-	-	-
Han ethnicity (%)	100%	100%	NS
Proteinuria (g/24 h)	1.28 ± 0.38	6.75 ± 0.51	$P<0.01^{**}$
Systolic blood pressure (mmHg)	101.33 ± 5.37	169.31 ± 8.28	$P<0.05^{*}$
Diastolic blood pressure (mmHg)	77.23 ± 8.53	106.25 ± 6.97	$P<0.05^{*}$

The values are presented as the mean ± SEM. Statistical analyses were performed using one-way ANOVA. sPE, severe preeclampsia; NS denotes $P>0.05$; *, $P<0.05$; **, $P<0.01$.

#, The control placentas were from patients of cervical incompetency without infection or other possible diseases which would increase the risk of abortion or affecting placentation. The sPE patients involved in the current study did not present complications.

Table S1 b. Demographic and clinical characteristics of the normal pregnant women and sPE patients of second trimester enrolled in this study [#].

Characteristics	Control (n = 12)	sPE (n = 10)	*P* value
Maternal age (years)	29.91 ±3.28	33.56 ±4.37	NS
Preeclampsia onset (weeks)	None	23.36 ±4.32	-
Gestational age at delivery (weeks)	38.33 ±0.51	35.15 ±1.12	NS
Primiparae (%)	89.56%	82.37%	NS
Birth weight (g)	3895.45 ±384.89	2667.58 ±567.30	$P<0.05$*
Female fetus (n)	43.52%	45.56%	NS
Han ethnicity (%)	100%	100%	NS
Proteinuria (g/24 h)	2.31 ±0.38	5.64 ±0.79	$P<0.05^{**}$
Systolic blood pressure (mmHg)	116.67 ±7.32	156.53 ±5.36	$P<0.05^{*}$
Diastolic blood pressure (mmHg)	82.67 ±6.87	101.32 ±8.76	$P<0.05^{*}$

The values are presented as the mean ±SEM. Statistical analyses were performed using one-way ANOVA. sPE, severe preeclampsia; NS denotes $P>0.05$; *, $P<0.05$; **, $P<0.01$.

[#], The control placentas were from patients of cervical incompetency without infection or other possible diseases which would increase the risk of abortion or affecting placentation. The sPE patients involved in the current study did not present complications.

Development • Supplementary information

Table S2. Antibodies used for IF, IHC and western blot.

Antibody	Source (Catalog no.)	Specificity	Application
HLA-G	Santa Cruz (sc-21799)	Extravillous trophoblast cells	IF, IHC, WB
Integrinα5	Santa Cruz (sc-10729)	Extravillous trophoblast cells	WB
CK7	Invitrogen (180234)	Epithelial cells, including trophoblast cells	IF, IHC
Vimentin	Invitrogen (180052)	Stromal cells and villous mesenchymal cells	IF
α-SMA	Abcam (ab5694)	Vascular smooth muscle cells	IF
PLAC8	ATLAS (HPA040465)	Extravillous trophoblast cells	IF, IHC, WB
CD34	Leica Biosystems (NCL-L-END)	Microvascular endothelial cells	IF

Table S3. Combinations of antibodies used in dual IF staining.

Sets	First primary (rabbit anti-human) and dilution	Second primary (mouse anti-human) And dilution
1	PLAC8 (1:150)	CK7 (1:100)
2	PLAC8 (1:150)	HLA-G (1:200)
3	PLAC8 (1:150)	Vimentin (1:200)
4	PLAC8 (1:150)	CD34 (1:200)
5	α-SMA (1:200)	HLA-G (1:200)

Development • Supplementary information

Table S4. Differentially expressed genes in HTR8/SVneo cells with or without PLAC8 overexpression.

Click here to Download Table S4

Development • Supplementary information

Supplementary movie

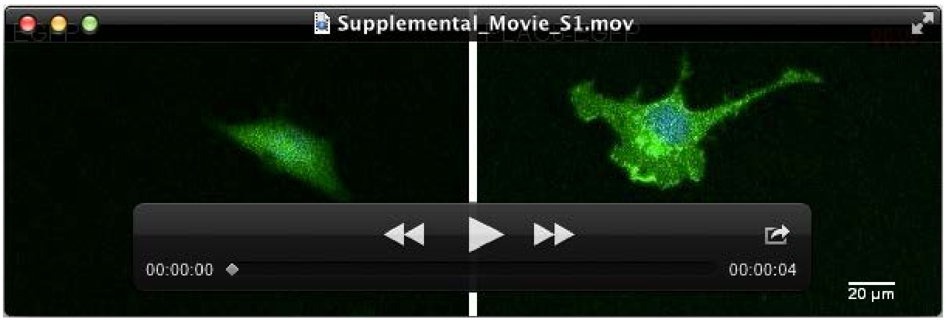

Movie S1. Time-lapse imaging showing migration of HTR8/SVneo cells with stable transfection of GFPandPLAC8-GFP (still images in Figure 7A, upper panel)

Development • Supplementary information

Supplementary Materials and Methods

Whole mount IF assay: For whole-mount fluorescent IHC, the tissues were fixed using 4% PFA and sequentially incubated with 10% FBS, primary antibody at 4 ℃ for 2 days, secondary antibody and DAPI. The appropriate concentrations of rabbit and mouse IgGs that were equivalent to the corresponding primary antibodies were applied to negative control sections to control for non-specific staining. Images were captured on a Carl Zeiss LSM 780 confocal laser-scanning microscope.

***In situ* hybridization:** Frozen sections were fixed in 4% PFA. Hybridization was performed at 55 ℃ in a hybridization buffer containing 200 mM NaCl, 13 mM Tris, 5 mM NaH_2PO_4, 5 mM Na_2HPO_4, 5 mM EDTA, 50% formamide, 10% dextran sulfate, 1 mg/ml salmon sperm DNA, 2% BSA and the DIG-labeled cRNA probes for*PLAC8*. The sections were washed twice at 55 ℃ in the post-hybridization buffer (1×SSC, 50% formamide, and 0.1% Tween-20) and twice in buffer I (150 mM NaCl and 100 mM Tris, pH 7.5). The sections were blocked in 2% blocking reagent (Roche Diagnostics, Mannheim, Germany) for 1 hour and incubated with a 1:2,000 dilution of the anti-DIG antibody (Roche) overnight at 4 ℃. After four washes in buffer I, the sections were rinsed in buffer III (100 mM NaCl, 50 mM $MgCl_2$, and 100 mM Tris, pH 9.5), and the signals were developed before being counterstained with nuclear fast red.

Cell line, RNA interference (RNAi) and PLAC8 overexpression: HTR8/SVneo cells were grown in RPMI 1640 medium (SH30809, Hyclone, Thermo Fisher Scientific Inc.) supplemented with 10% FBS (Gibco BRL, Carlsbad, CA), 100 units/ml penicillin and 100 μg/ml streptomycin. For PLAC8 knockdown, HTR8/SVneo cells were transfected with 100 nM*PLAC8* siRNA (5’-GGUGGUCGUUGUGACCCAACCUGGA-3’; and 5’-CAAAUCAAGAGAGAUAU CAACAGAA-3’, Invitrogen, MD; GenBank ID for PLAC8: NM_001130716.1), a universal negative control siRNA (Invitrogen) or a fluorescein isothiocyanate (FITC)-labeled positive control siRNA (Invitrogen) with Lipofectamine 2000 transfection reagent (Invitrogen, Carlsbad, CA), according to the manufacturer’s instructions. Both siRNAs for *PLAC8* specifically targeted the homologous region of the three known transcript variants of the human *PLAC8* gene. For overexpression, the full-length EGFP-tagged PLAC8 was subcloned into the pIRES2-EGFP vector. HTR8/SVneo cells were transfected with the pIRES2-EGFP EV or the pIRES2-PLAC8-EGFP vector and were then screened by flow cytometry to generate stably transfected cell lines.

Development • Supplementary information

Matrigel invasion and transwell migration assay: Invasion was performed in matrigel-coated transwell inserts (6.5 mm, Costar, Cambridge, UK) containing polycarbonate filters with 8-μm pores. Briefly, the inserts were pre-coated with 50 μl of matrigel matrix (1 mg/ml; the inserts were not pre-coated with matrigel in the cell migration assay), and 1×10^5 cells were plated in the upper chamber. After 24h incubation, the cells on the upper side of each insert were removed with a cotton swab. The inserts were fixed in methanol and stained with hematoxylin and eosin. The invaded (or migratory) cells that were attached to the other side of the insert were photographed under a light microscope (Nikon Eclipse Ti, Tokyo, Japan). The numbers of cells in 10 randomly selected fields were counted at a magnification of 200× by an observer who was blinded to the conditions. The results represent the mean values of invasion/migration percentage (%) ± SEM compared with the control.

Wound healing assay: Cells were cultured on a 6-well plate, and when the cells were completely confluent, the monolayer was manually scraped with a 1-ml pipette tip and washed with PBS to remove the non-adherent cells. Wound scraping was considered as time 0h, and the cells were photographed when the scratch was made; the same position was photographed every 6 hours. For the rescue experiment, *PLAC8* siRNAs were transfected into the stably transfected HTR8/SVneo cells (pIRES2-EGFP or pIRES2-PLAC8-EGFP) with Lipofectamine 2000 and Opti-MEM (Invitrogen, Carlsbad, CA) 24 h before plating. The wound-healing efficiency was determined by measuring the area of confluent cells and was expressed as a percentage of the relative healing area.

RNA sequencing: Transcriptome change in trophoblast cell line of stable PLAC8-EGFP overexpression was investigated by high-throughput sequencing assay with the Illumina HiSeq 2500 (Homo sapiens) platform. HTR8/SVneo with stable PLAC8-EGFP overexpression and corresponding negative control (HTR8/SVneo cells with EGFP overexpression), each with two repeats, were assigned for sequencing assay. Corresponding sequencing results have been deposited at the GEO database (GEO reviewer token: kxgrwomcdrozbcd to GSE105783; website link, https://www.ncbi.nlm.nih.gov/geo/query/acc.cgi?acc=GSE105783).

Development • Supplementary information
